# Ethnic inequalities in clozapine use among people with treatment-resistant schizophrenia: a retrospective cohort study using data from electronic clinical records

**DOI:** 10.1007/s00127-022-02257-3

**Published:** 2022-03-04

**Authors:** Daniela Fonseca de Freitas, India Patel, Giouliana Kadra-Scalzo, Megan Pritchard, Hitesh Shetty, Matthew Broadbent, Rashmi Patel, Johnny Downs, Aviv Segev, Mizanur Khondoker, James H. MacCabe, Kamaldeep Bhui, Richard D. Hayes

**Affiliations:** 1 Department of Psychological Medicine, Institute of Psychiatry, Psychology, and Neuroscience, King’s College London, 16 De Crespigny Park, Denmark Hill, London, SE5 8AF UK; 2 Department of Psychiatry, University of Oxford, Oxford, UK; 3 South London and Maudsley NHS Foundation Trust, London, UK; 4 Department of Psychiatry, Sackler Faculty of Medicine, Tel Aviv University, Tel Aviv, Israel; 5 Shalvata Mental Health Center, Hod Hasharon, Israel; 6 Norwich Medical School, University of East Anglia, Norwich, UK; 7 Nuffield Department of Primary Care Health Sciences, University of Oxford, Oxford, UK

**Keywords:** Refractory psychosis, Clozapine, Benign ethnic neutropenia, Black British, Asian British, Health inequalities

## Abstract

**Purpose:**

Clozapine is the most effective intervention for treatment-resistant schizophrenia (TRS). Several studies report ethnic disparities in clozapine treatment. However, few studies restrict analyses to TRS cohorts alone or address confounding by benign ethnic neutropenia. This study investigates ethnic equity in access to clozapine treatment for people with treatment-resistant schizophrenia spectrum disorder.

**Methods:**

A retrospective cohort study, using information from 11 years of clinical records (2007–2017) from the South London and Maudsley NHS Trust. We identified a cohort of service-users with TRS using a validated algorithm. We investigated associations between ethnicity and clozapine treatment, adjusting for sociodemographic factors, psychiatric multi-morbidity, substance misuse, neutropenia, and service-use.

**Results:**

Among 2239 cases of TRS, Black service-users were less likely to be receive clozapine compared with White British service-users after adjusting for confounders (Black African aOR = 0.49, 95% CI [0.33, 0.74], *p* = 0.001; Black Caribbean aOR = 0.64, 95% CI [0.43, 0.93], *p* = 0.019; Black British aOR = 0.61, 95% CI [0.41, 0.91], *p* = 0.016). It was additionally observed that neutropenia was not related to treatment with clozapine. Also, a detention under the Mental Health Act was negatively associated clozapine receipt, suggesting people with TRS who were detained are less likely to be treated with clozapine.

**Conclusion:**

Black service-users with TRS were less likely to receive clozapine than White British service-users. Considering the protective effect of treatment with clozapine, these inequities may place Black service-users at higher risk for hospital admissions and mortality.

**Supplementary Information:**

The online version contains supplementary material available at 10.1007/s00127-022-02257-3.

## Background

Studies show ethnic inequalities in healthcare. For instance, ethnic minorities received poorer analgesic care [1] and reported worse experiences in maternal and in cancer care [2, 3]. Among people with a common mental disorder, compared to White British people, ethnic minorities are less likely to receive effective treatment [4], less likely to be referred to secondary care by their general practitioner (GP), and more likely to be referred to inpatient or emergence services [5]. A similar pattern in the pathway to care in psychosis is observed, with some ethnic minority groups having lower GP involvement and more compulsory admission [6]. Moreover, Black people are less likely to receive psychotherapies (cognitive behaviour/family therapy), and are more likely to receive a long-acting injectable antipsychotic [7, 8].

Treatment-resistant schizophrenia (TRS) is defined as a failure to respond to two trials of antipsychotics with an adequate dose [9] and affects up to a third of people living with schizophrenia [10]. Clozapine is the recommended antipsychotic for TRS [11], producing superior outcomes than other antipsychotics in symptom response, user satisfaction [12, 13], hospitalisation [14, 15], and mortality [16, 17]. Some studies, including a systematic review, show that Black people are less likely to receive this drug [7, 16, 18–25]. However, not all studies report ethnic differences in clozapine prescription [26, 27], and mixed findings are observed even in studies conducted in the same region, such as London [16, 21, 26, 27].

Clinicians and minority ethnic service-users may resist clozapine treatment because of benign ethnic neutropenia (BEN). This is a condition of low baseline level of white cells that is more common in people of African, Arabian, and Mediterranean background [28, 29]. Clozapine-induced lowering of white cells (agranulocytosis) and related death is extremely rare [30], and the presence of BEN does not necessarily prevent the treatment with clozapine [28]. However, clozapine can only be prescribed after haematological consultation to assess neutropenia and a white cell count monitoring protocol. The presence of BEN could act as a barrier to clozapine treatment in Black people, but this has not been empirically tested. Other limitations of previous studies include the lack of a cohort of people with TRS (with the risk of confounding by indication) [23, 26], or cohorts of only inpatients or outpatients (restricting the generalisability of findings) [21, 26].

This study addresses these limitations by investigating ethnic disparities in the prescription of clozapine in a cohort of people with TRS, while adjusting for several potential confounders, including sociodemographic information, psychiatric multi-morbidity, substance misuse, service-use, and neutropenia. Our hypothesis is that ethnic minority services-users, specially Black people, are less likely to be treated with clozapine than their White British counterparts.

## Methods

### Setting and data sources

In this retrospective cohort study, we used data from the electronic health records (EHRs) of the South London and Maudsley (SLaM) National Health Service (NHS) Foundation Trust. SLaM's catchment area comprises four London boroughs (Southwark, Lewisham, Lambeth, and Croydon), with a population of 1.3 M (2018 estimates). Access to the information to SLaM's clinical records is made via the Clinical Record Interactive Search system (CRIS) established in 2007–2008 [31, 32]. At the time of writing, CRIS enabled access to the de-identified information, in the free-text and structured fields, of over 400,000 service-users. Multiple Natural Language Processing (NLP) applications are used to retrieve information on the free-text fields [31–34]. These applications outperform simple keyword searches, by identifying conditions of interest while distinguishing them from situations where related words are mentioned but are not the condition of interest [32, 34].

Moreover, CRIS provides several data linkages to national registries, including the Zaponex Treatment Access System (ZTAS) [16]. The ZTAS registry is the monitoring system for clozapine treatment used at SLaM (due to an exclusive license for the use of Zaponex). The monitoring system aims to prevent negative outcomes of clozapine-induced agranulocytosis and record-related relevant conditions, such as BEN. At the time of project conception, CRIS had a linkage enabling access to ZTAS data up to 31/03/2016.

CRIS was established under a robust data protection and governance framework and received approval from the Oxford C Research Ethics Committee (18/SC/0372) to be used as a de-identified dataset for secondary data analysis [31, 32]. This project was approved by the service user-led CRIS oversight committee.

### Sample inclusion criteria

The sample comprises SLaM service-users who met the following inclusion criteria: (1) had a primary diagnosis of a schizophrenia spectrum disorder (ICD-10: F20–F29), (2) were taking antipsychotics between 1 January 2007 and 31 December 2017, (3) lived in the SLaM catchment or were homeless, (4) were 18 years or older at the time of that first antipsychotic after 2007, and (5) met a proxy definition for treatment resistance, via a validated algorithm.

### Ascertainment of treatment resistance

TRS was identified in an automated way, via the development and validation of several algorithms against a gold-standard, manually coded, dataset of TRS cases in a previous study [35]. In line with recommendations [9, 11], the coding rules for the ascertainment of TRS for the gold-standard dataset were evidence of (1) failure to respond to two 6-week trials of different antipsychotics or (2) clozapine prescription. Failure of response was assumed when the switch of the antipsychotic was not a result of non-adherence or adverse side-effects.

The algorithms developed to identify TRS in a large dataset were based on an automatic coding of trials of antipsychotic treatment and episodes of hospitalisation [11, 16]. An antipsychotic trial was defined by evidence of at least two prescriptions of the same antipsychotic, at least 6 weeks apart. To ensure that we did not exclude polypharmacy, which could be a switch from one antipsychotic to another, the first prescription of a new antipsychotic could take place at any point after the prescription of the previous and not only 6 weeks after. We selected the algorithm which performed best based on its precision (i.e., positive predictive value) and recall (i.e., sensitivity). More information on the algorithms is presented in the Supplementary Material Table S1. With the selected algorithm, the proxy definition for TRS was evidence of six trials of different antipsychotic drugs, clozapine prescription, or ZTAS registration. This algorithm provided good precision (84%) and recall (73%) against the gold-standard dataset. TRS date was assigned to the earliest occurrence of any of the three criteria for TRS.

### Measures

Clozapine prescription between 1 January 2007 and 31 December 2017 (the study’s outcome), as well as information regarding the antipsychotic treatments used in the TRS algorithms, was retrieved using NLP applications [32, 33]. The applications identified antipsychotics by their generic and brand names. To increase reliability, antipsychotics had to be accompanied by dosage, but no minimum dose was established.

Ethnicity was based on the 16 ethnic categories used by the NHS, which were aggregated into eight categories due to small sample sizes. These were: (1) White British; (2) Other White [Irish and Other White background]; (3) Black African [Black African and White and Black African]; (4) Black Caribbean [Black Caribbean and White and Black Caribbean]; (5) Black British or Other Black background; (6) South Asian [Bangladeshi, Indian and Pakistani]; (7) Asian British or Other Asian [Chinese, White and Asian, and Other Asian background]; and (8) Other Ethnicity [Other Mixed background and any Other ethnicity].

Potential confounders included sociodemographic information, psychiatric diagnoses, substance misuse, other clinical and functional status factors, evidence of neutropenia (including benign ethnic neutropenia), and information regarding service-use. Sociodemographic confounders included gender, age at the date of TRS, homelessness ever before TRS, and neighbourhood deprivation. The latter was the index of multiple deprivation of the English Indices of Deprivation [36] assigned to the service-user’s address at TRS date.

Psychiatric diagnoses included the schizophrenia spectrum diagnosis and psychiatric comorbidities. Considering the possibility of multiple diagnoses within the schizophrenia spectrum being mentioned in clinical records at TRS date, the diagnoses were categorised according to the highest hierarchy: schizoaffective disorder (F25), schizophrenia (F20) or other diagnoses (F21–F24, F28–F29). Psychiatric comorbidities reported at any time before the TRS date comprised: (1) developmental disorders [development disorder, including autism (F80–F84), intellectual disability (F70–F79) or attention (F90)]; (2) anxiety-related disorders [anxiety (F40–F41), obsessive–compulsive (F42–F43), and post-traumatic stress (F43.1)], (3) bipolar (F30–F31, F34), (4) depression or other mood disorders excluding bipolar (F32–F39), and (5) personality disorder (F60–F61).

Evidence of substance misuse comprised having ever been diagnosed with any substance use-related disorder (F10–F14, F16, F18–F19) and previous use of cannabis. Cannabis use was identified using an NLP application [33, 37]. Other clinical, social, and functional factors were assessed using the 12 items of the Health of the Nation Outcomes Scales (HoNOS) [38]. Information was retrieved from the closest HoNOS to TRS date, but within one year before. The ratings were collapsed from five categories into 0 (not a problem) and 1 (a problem of any severity).

Given that a proportion of patients with neutropenia will be classified as having benign ethnic neutropenia (BEN), in this study, patients with BEN were included together with other patients who had evidence of neutropenia. Neutropenia was assumed when there were low neutrophil counts (< 2.2 × 10^9^/L) in SLaM or ZTAS records, on two separate occasions at least 1 month apart. Information regarding BEN was retrieved from ZTAS sociodemographic information; thus, data on BEN were only available for service-users with records on ZTAS. No evidence of BEN or neutropenia, or no information on neutrophil counts, was coded as lack of evidence of neutropenia. Information on evidence of neutropenia was retrieved up to the point of clozapine prescription or the end of the observation window.

Service-use measures comprised: number of days of hospitalisation within the 3 months prior to TRS date; number of days on which service-users had face-to-face contacts with outpatient teams in the 3 months prior to the TRS date; and if the service-users were detained to receive care under the Mental Health Act 1983 [39] at any time before TRS. We included civil involuntary hospitalisation under Part 2 of the Act, detention under police use of power, and forensic detentions under Part 3 of the Act.

### Statistical analysis

Descriptive information and the relationships between ethnicity and the covariates are presented along with chi-squared analyses or analyses of variance (ANOVAs). Logistic regression analyses were performed to investigate the association between ethnicity and clozapine prescription, controlling for each category of possible confounders. A fully adjusted model is presented, considering the number of covariates. Statistical analyses were conducted using STATA 15 [40].

## Results

### Participants

The sample comprises 2239 people meeting the inclusion criteria for TRS. Only 0.3% of participants were excluded because of missing ethnicity data. Service-users’ age was 18–90 years (*M* = 40.9, SD = 12.6) and 64% were men. Ethnic composition was: 32% were White British; 18% were Black Caribbean; 17% were Black British; 16% were Black African; 7% were from Other White background; 4% were Asian British, and 3% were South Asian (Table 1). The proportion of people across the various ethnic groups in the algorithm based TRS sample was similar to one observed in the manually coded TRS cohort.

**Table 1:** Demographic and clinical characteristics of people with treatment-resistant schizophrenia

Sample characteristics	Total: *n* (% of total sample)	Prescribed clozapine: *n* (% per characteristic)
Total	2239 (100)	1837 (82.1)
Ethnicity		
White British	719 (32.1)	628 (87.3)
Black Caribbean	391 (17.5)	303 (77.5)
Black British/other Black background	371 (16.6)	296 (79.8)
Black African	354 (15.8)	267 (75.4)
Other White background	150 (6.7)	128 (85.3)
Other ethnic background	109 (4.9)	92 (84.4)
Asian British/other Asian background	84 (3.8)	69 (82.1)
South Asian	61 (2.7)	54 (88.5)
Sociodemographic factors		
Gender		
Men	1438 (64.2)	1221 (84.9)
Women	801 (35.8)	616 (76.9)
Age: *M* (SD)	40.9 (16.6)	40.2 (12.3)
Ever homeless before TRS date	214 (9.6)	130 (60.8)
Neighbourhood deprivation score (6.7% missing)^a^: *M* (SD)	31.6 (9.2)	31.5 (9.3)
Primary diagnosis		
Schizophrenia	1611 (72.0)	1350 (83.8)
Schizoaffective disorder	412 (18.4)	300 (72.8)
Other prolonged psychosis	216 (9.7)	187 (86.6)
Psychiatric comorbidities		
Developmental disorder		
No	2083 (93.0)	1771 (82.1)
Yes	156 (6.8)	126 (80.8)
Anxiety-related disorder		
No	2124 (94.9)	1752 (82.5)
Yes	115 (5.1)	85 (73.9)
Bipolar disorder		
No	1891 (84.5)	1612 (85.3)
Yes	348 (15.5)	225 (64.7)
Depression or other mood disorder (excluding bipolar)		
No	1889 (84.4)	1568 (83.0)
Yes	350 (15.6)	269 (76.9)
Personality disorder		
No	1961 (87.6)	1631 (83.2)
Yes	278 (12.4)	206 (74.1)
Substance misuse		
Any substance use-related disorder		
No	1954 (87.3)	1639 (83.9)
Yes	285 (12.7)	198 (69.5)
Evidence of cannabis use before TRS date		
No	846 (37.8)	751 (88.8)
Yes	1393 (62.2)	1086 (78.0)
The Health of the Nation Outcome Scale (HoNOS)		
Behaviour and symptoms		
Overactive, aggressive behaviour (26.80% missing)		
Not a problem	749 (45.7)	612 (81.7)
Problem	889 (54.3)	668 (75.1)
Hallucinations and delusions (27.0% missing)		
Not a problem	309 (18.9)	237 (76.7)
Problem	1325 (98.1)	1041 (78.6)
Depressed mood (27.1% missing)		
Not a problem	789 (48.3)	611 (77.4)
Problem	843 (51.7)	665 (78.9)
Non-accidental self-injury (26.3% missing)		
Not a problem	1430 (87.4)	1109 (77.6)
Problem	206 (12.6)	169 (82.1)
Problem drinking or drug-taking (27.4% missing)		
Not a problem	1119 (68.9)	890 (79.5)
Problem	506 (31.1)	383 (75.7)
Cognitive problems (27.1% missing)		
Not a problem	819 (50.1)	639 (78.0)
Problem	815 (49.9)	637 (78.2)
Physical illness or disability problems (27.1% missing)^b^		
Not a problem	966 (59.1)	770 (79.7)
Problem	667 (40.9)	505 (75.7)
Impairment, social and functional status (HoNOS)		
Social relationships (27.4% missing)		
Not a problem	511 (31.4)	406 (79.5)
Problem	1115 (68.6)	866 (77.7)
Activities of daily living (ADLs) (27.6% missing)		
Not a problem	613 (37.8)	480 (78.3)
Problem	1009 (62.2)	791 (78.4)
Living conditions (29.6% missing)		
Not a problem	923 (58.6)	741 (80.3)
Problem	653 (41.4)	497 (76.1)
Occupational and recreational activities (29.2% missing)		
Not a problem	618 (39.0)	488 (79.0)
Problems	967 (61.0)	757 (78.3)
Evidence of neutropenia or BEN		
No	2078 (92.8)	1712 (82.4)
Yes	161 (7.2)	125 (77.6)
Service-use		
Number of days of face-to-face contact with SLaM services in the 3 months before TRS date (Md 5, IQR 2–9, range 0–91)		
0	294 (13.1)	267 (90.8)
1–7	1235 (55.2)	1014 (82.1)
8–14	462 (20.6)	366 (79.2)
15 and above	248 (11.1)	190 (76.6)
Number of SLaM days in hospitalisation the 3 months before TRS date (Md 0, IQR 0–44, range 0–91)		
0	1152 (51.5)	1003 (87.1)
1–29	401 (17.9)	289 (72.1)
30–59	216 (9.7)	166 (76.9)
60 and above	470 (21.0)	379 (80.6)
Involuntary care under the MHA before TRS date		
Part 2—civil detention		
No	1102 (49.2)	1029 (93.4)
Yes	1137 (50.8)	808 (71.1)
Police detention		
No	1876 (83.8)	1611 (85.9)
Yes	363 (16.2)	226 (62.3)
Part 3—forensic detention		
No	2055 (91.8)	1600 (82.7)
Yes	184 (8.2)	138 (75.0)

### Sociodemographic and clinical characteristics of the TRS cohort by ethnicity

We observed significant ethnic differences in the sociodemographic and clinical characteristics of people with TRS (Table 2). To mention a few, Black African and Black British people were aged between 36 and 37, whereas the mean age for Black Caribbean, White and Asian people was higher (41–43 years). Homelessness was more common in the Other White (16%) and Black African (13%) people.

**Table 2:** Demographic and clinical characteristics among ethnic groups with treatment-resistant schizophrenia

Sample characteristics	White British *n* (%)	Black Caribbean *n* (%)	Black British/other Black background *n* (%)	Black African *n* (%)	Other White background *n* (%)	Other ethnicity *n* (%)	Asian British/other Asian background *n* (%)	South Asian *n* (%)	*p* value
Sociodemographic factors									
Gender—women	250 (34.8)	143 (36.6)	119 (32.1)	147 (41.5)	49 (32.7)	35 (32.1)	32 (38.1)	26 (42.6)	*χ*^2^(7) = 10.44, *p* = 0.165
Age: *M* (SD)	43.6 (13.2)	44.0 (11.9)	36.1 (10.1)	37.4 (11.7)	41.0 (13.6)	37.5 (12.0)	41.8 (13.3)	42.2 (12.6)	*F*(7) = 22.54, *p* < 0.001
Ever homeless before TRS date	49 (6.8)	35 (9.0)	45 (12.3)	45 (12.9)	23 (15.5)	10 (9.2)	< 10%	< 5%	*χ*^2^(7) = 23.19, *p* = 0.002
Neighbourhood deprivation score: *M* (SD)	30.5 (9.9)	32.7 (8.6)	32.2 (8.6)	33.0 (8.7)	30.8 (10.0)	32.0 (8.2)	31.3 (8.9)	29.0 (8.6)	*F*(7) = 4.38, *p* < 0.001
Primary diagnosis									*χ*^2^(14) = 31.06, *p* = 0.005
Schizophrenia	573 (79.7)	307 (78.5)	294 (79.3)	261 (73.7)	120 (80.0)	82 (75.2)	62 (74.8)	55 (90.2)	
Schizoaffective disorder	125 (17.4)	76 (19.4)	65 (17.5)	73 (20.6)	26 (17.3)	22 (20.2)	19 (22.6)	< 10%	
Other prolonged psychosis	94 (13.1)	23 (5.9)	22 (5.9)	34 (9.6)	18 (12.0)	11 (10.1)	10 (11.9)	< 10%	
Psychiatric comorbidities									
Developmental disorders	48 (6.7)	27 (6.9)	33 (8.9)	23 (6.5)	13 (8.7)	< 10%	< 5%	< 5%	*χ*^2^(7) = 6.18, *p* = 0.519
Anxiety-related disorders	50 (7.0)	< 5%	17 (4.6)	15 (4.2)	11 (7.3)	< 10%	< 10%	< 5%	*χ*^2^(7) = 13.82, *p* = 0.054
Bipolar disorder	106 (14.7)	56 (14.3)	60 (16.2)	68 (19.2)	23 (15.3)	17 (15.6)	< 10%	10 (16.4)	*χ*^2^(7) = 6.89, *p* = 0.441
Depression or other mood disorders (excl. bipolar)	132 (18.4)	43 (11.0)	61 (16.4)	55 (15.5)	32 (21.3)	10 (9.2)	11 (13.1)	< 10%	*χ*^2^(7) = 19.72, *p* = 0.006
Personality disorder	123 (17.1)	40 (10.2)	45 (12.1)	29 (8.2)	22 (14.7)	< 10%	< 10%	< 10%	*χ*^2^(7) = 29.24, *p* < 0.001
Substance misuse									
Any substance use-related disorder	103 (14.3)	58 (14.8)	52 (14.0)	34 (9.6)	21 (14.0)	< 10%	< 10%	< 5%	*χ*^2^(7) = 17.27, *p* = 0.016
Evidence of cannabis use before TRS date	394 (54.8)	275 (70.3)	282 (76.0)	219 (61.9)	89 (59.3)	65 (59.6)	44 (52.4)	25 (41.0)	*χ*^2^(7) = 73.83, *p* < 0.001
The Health of the Nation Outcome Scale (HoNOS)									
Behaviour and symptoms									
Overactive, aggressive behaviour	250 (52.1)	152 (51.4)	167 (58.4)	164 (55.0)	51 (54.3)	48 (59.3)	33 (55.9)	24 (54.6)	*χ*^2^(7) = 4.85, *p* = 0.679
Non-accidental self-injury	81 (16.9)	22 (7.4)	33 (11.6)	23 (7.7)	17 (18.1)	12 (14.8)	10 (17.0)	< 20%	*χ*^2^(7) = 27.04, *p* < 0.001
Problem with drinking or drug-taking	155 (32.4)	91 (30.9)	102 (36.4)	81 (27.5)	28 (30.11)	26 (32.1)	13 (22.0)	10 (22.7)	*χ*^2^(7) = 9.71, *p* = 0.205
Hallucinations and delusions	389 (81.2)	224 (75.7)	228 (79.7)	247 (83.7)	73 (77.7)	73 (90.1)	52 (88.1)	39 (88.6)	*χ*^2^(7) = 15.93, *p* = 0.026
Depressed mood	274 (57.2)	135 (45.6)	130 (45.8)	131 (44.4)	54 (57.5)	61 (75.3)	39 (66.1)	19 (43.2)	*χ*^2^(7) = 45.98, *p* < 0.001
Impairment, social and functional status									
Cognitive problems	242 (50.6)	150 (50.9)	140 (49.0)	133 (44.8)	48 (51.1)	44 (54.3)	37 (62.7)	21 (47.7)	*χ*^2^(7) = 8.06, *p* = 0.327
Physical illness or disability problems	218 (45.8)	129 (43.7)	97 (33.9)	115 (38.6)	42 (44.7)	23 (28.4)	25 (42.4)	18 (40.9)	*χ*^2^(7) = 17.98, *p* = 0.012
Social relationships	327 (68.6)	183 (62.2)	200 (70.7)	213 (72.2)	63 (67.7)	55 (67.9)	42 (71.2)	32 (72.7)	*χ*^2^(7) = 8.43, *p* = 0.296
Activities of daily living	318 (66.5)	186 (63.5)	177 (62.3)	170 (58.2)	55 (59.1)	48 (60.0)	32 (55.2)	23 (52.3)	*χ*^2^(7) = 9.56, *p* = 0.214
Living conditions	178 (38.0)	120 (42.7)	125 (46.1)	123 (43.0)	40 (44.9)	30 (38.0)	28 (48.3)	< 25%	*χ*^2^(7) = 14.69, *p* = 0.040
Occupational and recreational activities	278 (58.7)	174 (61.7)	171 (63.6)	174 (60.2)	61 (67.8)	52 (65.0)	37 (63.8)	20 (46.5)	*χ*^2^(7) = 8.24, *p* = 0.312
Evidence of neutropenia or BEN	15 (2.1)	29 (7.4)	46 (12.4)	54 (15.3)	< 5%	< 10%	< 5%	< 5%	*χ*^2^(7) = 86.34, *p* < 0.001
Service- use									
Number of days of face-to-face contact with SLaM services in the 3 months before TRS date									*χ*^2^(21) = 20.83, *p* = 0.469
0	92 (12.8)	58 (14.8)	51 (13.8)	45 (12.7)	24 (16.0)	< 10%	< 10%	< 15%	
1–7	393 (54.7)	220 (56.3)	211 (56.9)	176 (49.7)	83 (55.3)	70 (64.2)	47 (56.0)	35 (57.4)	
8–14	147 (20.5)	76 (19.4)	73 (19.7)	90 (25.4)	24 (16.0)	22 (20.2)	20 (23.8)	10 (16.4)	
15 and above	87 (12.1)	37 (9.5)	36 (9.7)	43 (12.2)	19 (12.7)	10 (9.2)	< 15%	< 15%	
Number of SLaM days in hospitalisation in the 3 months before TRS date									*χ*^2^(21) = 58.03, *p* < 0.001
0	425 (59.1)	199 (50.9)	163 (43.9)	148 (41.8)	84 (56.0)	58 (53.2)	42 (50.0)	33 (54.1)	
1–29	119 (16.6)	59 (15.1)	68 (18.3)	72 (20.3)	28 (18.7)	21 (19.3)	20 (23.8)	14 (23.0)	
30–59	64 (8.9)	41 (10.5)	40 (10.8)	44 (12.4)	13 (8.7)	< 10%	< 5%	< 5%	
60 and above	111 (15.4)	92 (23.5)	100 (27.0)	90 (25.4)	25 (16.7)	22 (20.2)	18 (21.4)	12 (19.7)	
Involuntary care under the MHA before TRS date									
Part 2—civil detention	271 (37.7)	209 (53.5)	218 (58.8)	239 (67.5)	71 (47.3)	58 (53.2)	45 (53.6)	26 (42.6)	*χ*^2^(7) = 102.37, *p* < 0.001
Police detention	81 (11.3)	67 (17.1)	71 (19.1)	91 (25.7)	20 (13.3)	17 (15.6)	< 10%	< 15%	*χ*^2^(7) = 43.17, *p* < 0.001
Part 3—forensic detention	37 (5.2)	44 (11.3)	46 (12.4)	34 (9.6)	14 (9.3)	< 5%	< 5%	< 5%	*χ*^2^(7) = 31.19, *p* < 0.001

Note: In cells where the number of people was smaller than 10, we present the closest % in multiples of 5

White British people were more frequently diagnosed with comorbid mood (excluding bipolar) and personality disorders (18% and 17%, respectively). A substance use disorder was less frequent in South Asian people (< 5%). Cannabis use was less common in White British (55%) and South Asian people (41%) and more common in the Black Caribbean (70%) and Black British people (76%). According to HoNOS ratings, hallucinations were less common in Black Caribbean people (76%), and depressed mood was more common in Other ethnicity (75%).

As expected, there were major differences in the proportion of people with evidence of neutropenia (including BEN), with the highest levels among Black African (15%), Black British (12%), Black Caribbean people (7%) and lower in the White British (2%). Analyses revealed a 3 to 8 fold higher relative odds for neutropenia among these ethnic groups, compared with the White British: OR_Black African_ = 8.45, 95% CI [4.69, 15.20], *p* < 0.001; OR_Black British_ = 6.64, 95% CI [3.65, 12.07], *p* < 0.001, OR_Black Caribbean_ = 3.76, 95% CI [1.99, 7.10], *p* < 0.001. The relative risk was not meaningfully calculated for other ethnic groups due to the small number of people with neutropenia (Table 2).

An episode of involuntary hospitalisation under the MHA Part 2 was more frequent among Black African (66%) and Black British (59%), than White British people (38%). Police detention was more common among Black African (26%) and less frequent in White British people (11%).

### Ethnicity and clozapine treatment

In this cohort of people with TRS, 75% of the Black African people, 89% of the South Asian and 87% of White British people received clozapine (Table 1). Results from the logistic regression analyses (Table 3) show that when compared to the White British people (reference), Black African people were half as likely to receive clozapine (aOR = 0.49, 95% CI [0.33, 0.74], *p* = 0.001). Black Caribbean and Black British or Other Black people also less likely to receive clozapine (aOR_Black Caribbean_ = 0.64, 95% CI [0.43, 0.93], *p* = 0.019; aOR_Black British/Black Other_ = 0.61, 95% CI [0.41, 0.91], *p* = 0.016). These results were adjusted for sociodemographics, psychiatric diagnoses, substance misuse, evidence of neutropenia, and type and frequency of service-use. No significant differences were observed for other ethnicities (Other White background or Asian background and any Other ethnicity). In the models adjusting only for the HoNOS items regarding symptoms and impairment, which due to substantial missing data were not included in the fully adjusted model, there were lower rates of clozapine treatment among Black African and Black Caribbean people. Sensitivity analyses, restricting the cohort to people who met the proxy criteria for TRS before the end date of the CRIS data linkage with ZTAS (31/03/2016), revealed similar findings to the fully adjusted model with the all cohort (results on Supplementary Material, Table S2).

**Table 3:** Ethnicity and odds of clozapine prescription in a cohort of people with TRS

Ethnicity	Odds ratio	[95% CI]	*p* value
Crude model (*N* = 2239)			
White British	Ref		
Black Caribbean	0.50	[0.36, 0.70]	< 0.001
Black British/other Black background	0.57	[0.41, 0.80]	0.001
Black African	0.44	[0.32, 0.61]	< 0.001
Other White background	0.84	[0.51, 1.39]	0.506
Other ethnic background	0.78	[0.44, 1.38]	0.397
Asian British/other Asian background	0.67	[0.37, 1.21]	0.185
South Asian	1.12	[0.49, 2.53]	0.789
Adjusted for sociodemographic factors^a^ (*N* = 2188)			
White British	Ref		
Black Caribbean	0.53	[0.38, 0.74]	< 0.001
Black British/other Black background	0.49	[0.33, 0.70]	< 0.001
Black African	0.38	[0.27, 0.55]	< 0.001
Other White background	0.86	[0.51, 1.47]	0.591
Other ethnic background	0.64	[0.36, 1.15]	0.135
Asian British/other Asian background	0.58	[0.32, 1.08]	0.088
South Asian	1.10	[0.45, 2.67]	0.833
Adjusted for diagnosis and comorbidities^b^ (*N* = 2239)			
White British	Ref		
Black Caribbean	0.45	[0.32, 0.64]	< 0.001
Black British/other Black background	0.55	[0.39, 0.78]	0.001
Black African	0.42	[0.31, 0.61]	< 0.001
Other White background	0.86	[0.51, 1.44]	0.571
Other ethnic background	0.72	[0.41, 1.29]	0.274
Asian British/other Asian background	0.59	[0.31, 1.08]	0.092
South Asian	1.05	[0.46, 2.41]	0.913
Adjusted for substance misuse^c^ (*N* = 2239)			
White British	Ref		
Black Caribbean	0.53	[0.38, 0.75]	< 0.001
Black British / other Black background	0.63	[0.45, 0.89]	0.009
Black African	0.44	[0.31, 0.61]	< 0.001
Other White background	0.85	[0.52, 1.43]	0.555
Other ethnic background	0.76	[0.43, 1.34]	0.341
Asian British/other Asian background	0.61	[0.34, 1.14]	0.121
South Asian	0.91	[0.40, 2.09]	0.833
Adjusted for behaviour and symptoms (HoNOS)^d^ (*N* = 1616)			
White British	Ref		
Black Caribbean	0.63	[0.44, 0.91]	0.013
Black British/other Black background	0.74	[0.51, 1.08]	0.125
Black African	0.57	[0.40, 0.82]	0.002
Other White background	0.81	[0.46, 1.41]	0.453
Other ethnic background	0.84	[0.46, 1.53]	0.569
Asian British/other Asian background	0.71	[0.36, 1.37]	0.305
South Asian	1.06	[0.45, 2.47]	0.898
Adjusted for impairment, social and functional problems (HoNOS)^e^ (*N* = 1537)			
White British	Ref		
Black Caribbean	0.62	[0.43, 0.90]	0.012
Black British/other Black background	0.69	[0.48, 1.03]	0.067
Black African	0.56	[0.39, 0.81]	0.002
Other White background	0.85	[0.47, 1.52]	0.576
Other ethnic background	0.74	[0.40, 1.36]	0.332
Asian British/other Asian background	0.71	[0.36, 1.38]	0.314
South Asian	1.17	[0.47, 2.89]	0.728
Adjusted for neutropenia or benign ethnic neutropenia (*N* = 2239)			
White British	Ref		
Black Caribbean	0.50	[0.36, 0.69]	< 0.001
Black British/other Black background	0.58	[0.41, 0.81]	0.006
Black African	0.45	[0.33, 0.63]	< 0.001
Other White background	0.84	[0.52, 1.40]	0.511
Other ethnic background	0.79	[0.45, 1.38]	0.407
Asian British/other Asian background	0.67	[0.37, 1.21]	0.187
South Asian	1.12	[0.49, 2.52]	0.793
Adjusted for service use^f^ (*N* = 2239)			
White British	Ref		
Black Caribbean	0.61	[0.43, 0.87]	0.006
Black British/other Black background	0.80	[0.56, 1.15]	0.233
Black African	0.71	[0.50, 1.00]	0.055
Other White background	1.05	[0.61, 1.80]	0.846
Other ethnic background	1.03	[0.57, 1.87]	0.922
Asian British/other Asian background	0.78	[0.41, 1.46]	0.435
South Asian	1.26	[0.53, 3.01]	0.593
Fully adjusted without HoNOS^g^ (*N* = 2188)			
White British	Ref		
Black Caribbean	0.64	[0.43, 0.93]	0.019
Black British/other Black background	0.61	[0.41, 0.91]	0.016
Black African	0.49	[0.33, 0.74]	0.001
Other White background	1.03	[0.58, 1.86]	0.904
Other ethnic background	0.65	[0.35, 1.23]	0.472
Asian British/other Asian background	0.62	[0.32, 1.23]	0.173
South Asian	1.09	[0.42, 2.85]	0.845

^a^Gender, age, homelessness and neighbourhood level of deprivation

^b^Main schizophrenia spectrum diagnosis and comorbid diagnoses (developmental, anxiety, bipolar, depressive, and personality disorders)

^c^Substance use-related disorder and cannabis use

^d^HoNOS items of behaviours and symptoms (overactive, self-injury, drinking or drinking or drug-taking, hallucinations, and depressed mood)

^e^HoNOS items of social and functional problems (cognitive, physical, relationships, activities of daily living, living conditions, and occupational problems)

^f^Number of days with face-to-face contact with services and in hospitalisation in the 3 months before TRS date, and involuntary care under the MHA

^g^All the variables on mentioned on a, b, c, and f. HoNOS variables (included under d and e) were excluded due to a large volume of missing data and its consequences to the statistical power of the analyses

It was also observed that neutropenia was not associated with treatment with clozapine; neither in a crude model (OR = 0.74, 95%CI [0.50, 1.09], *p* = 0.132), nor in the fully adjusted model (aOR = 0.82, 95%CI [0.51, 1.31], *p* = 0.415). Furthermore, people who had more than 30 days of hospitalisation, in the 3 months before the date they met proxy criteria for TRS, were more likely to be treated with clozapine (aOR_30–59 days_ = 1.78, 95%CI [1.15, 2.75], *p* = 0.010; aOR_60–90 days_ = 2.23, 95%CI [1.55, 3.20], *p* < 0.001), than those who were not hospitalised (results not shown in tables). Those who were admitted under the MHA, at any point before TRS date, were less likely to receive clozapine. This was observed for all types of detentions: hospitalisation initiated by clinical staff (aOR = 0.23, 95% CI [0.16, 0.33], *p* < 0.001), police conveying to a place of safety (aOR = 0.56, 95% CI [0.41, 0.75], *p* < 0.001), and forensic detention (aOR = 0.44, 95% CI [0.27, 0.71], *p* = 0.001).

## Discussion

We investigated potential ethnic differences in treatment with clozapine in a cohort of people with treatment-resistant schizophrenia. The results show that people of any Black background are less likely to receive clozapine than their White British counterparts. The differences were observed when controlling for other sociodemographic information, psychiatric comorbidities, substance misuse, evidence of neutropenia (including benign ethnic neutropenia), as well as frequency and type of service-use. No differences in clozapine treatment rates were observed between White British and people of any Asian background, Other White ethnicity, or any Other ethnicity.

Our results are consistent with the majority of studies, which report that minoritised ethnic people, particularly people from a Black background, are less likely to receive clozapine, the drug of choice for TRS [7, 20–23]. Our findings differ from a couple of studies [26, 27], but we used a cohort of people with TRS (which minimises confounding by indication) [26], and we have a much larger sample (which confers greater statistical power) [26, 27].

In this study, there were ethnic disparities in evidence of neutropenia (including benign ethnic neutropenia) confirming the greater prevalence of this condition among Black people [41]. The presence of neutropenia did not account for the ethnic differences in clozapine prescribing. This finding may be related to the fact that we measured mild neutropenia and not only severe neutropenia (< 1.5 × 10^9^/L). Thus, mild neutropenia, as well as benign ethnic neutropenia, should not prevent treatment with clozapine. This is in line with the recommended guidelines [28, 41].

Minoritised ethnic people more frequently received involuntary care via the use of the MHA. Higher detention rates are observed in multiple previous studies [6, 42] and may suggest more constrained relationships with health services [43]. Detention under the MHA could be associated with medical non-adherence, and this can be a barrier to clozapine treatment given the need for frequent monitoring [29, 44]. Thus, interventions may be needed to improve engagement among people who were detained under the MHA. Future research could investigate if such interventions result in better illness management and more treatment with clozapine. This might be effective if illness-related non-compliance with blood tests was the reason for non-prescription, but if people are, or were, detained on the MHA, they could and should receive this evidence-based recommended treatment.

The mechanisms that led to the reduced likelihood of Black people being treated with clozapine are not fully understood in this study. Disparities in physical health, not measured in the study, could be related to the observed inequalities. For instance, diabetes, hypertension and cardiovascular disease seem to be more prevalent among Black communities than White British people [45–47]. The potential impact of clozapine on these conditions may discourage clinicians and service-users from choosing this treatment [48], even though there may not be safe alternatives to clozapine, given that other antipsychotics with high efficacy, such as olanzapine, or zotepine, also have metabolic side-effects [49, 50]. Nonetheless, in a previous study in the USA, diabetes and cardiovascular illness had little association with clozapine treatment, and ethnic disparities persisted when controlling for those [24].

Having that limitation in mind, the findings of this study contribute to the accumulation of evidence regarding ethnic inequalities in care in the UK [2, 5, 8]. This evidence may indicate a pattern of structural racism in healthcare, which could be related to unconscious bias [51–53]. In the USA, clinicians' unconscious bias was associated with clinicians spending less time speaking with African–Americans, perceived lower warmth and friendliness, and lower quality of care [54]. An explanatory model proposes that negative stereotypes regarding ethnic minorities can arouse negative emotions in clinical staff, which then lead to clinical actions of neglect or harm (e.g., unnecessary invasive care) [52]. Notwithstanding, whether these systemic biases are prevalent in the UK, and their potential impact in healthcare, needs further study.

### Strengths and limitations

We used data from a large mental healthcare provider in the UK in an area whose population is very diverse (about 58.5% of residents are of an ethnic minority background) [55]. The service-users in the study should be representative of the study’s population, given the free access to care. The study's 11-year observation window allowed identifying a large sample of people with TRS and controlling for multiple confounders. Moreover, one of these was evidence of neutropenia, which has been reported as the potential major driver of ethnic disparities in clozapine treatment.

Some limitations include the use of an algorithm to identify TRS. The rules of the tested algorithms are not as fine-grained as those used to develop the manually coded gold-standard dataset. Moreover, it is likely that we are missing cases of TRS where there was no prescription of clozapine, given that we prioritised the precision of the algorithm. Unlike other cohort studies, a proportion of service-users were coded as having the outcome at the point of cohort entry, due to being treated with clozapine. Establishing a TRS cohort whose ascertainment criteria did not include clozapine would substantially reduce the sample size and compromise the achievement of this study aims. Given the focus on ethnicity, we believe the study’s cross-sectional design does not affect the reliability of the findings. There was substantial missing information in the HoNOS assessment, and preliminary analyses revealed that people whose TRS date corresponds to the date of clozapine or ZTAS registration had a higher proportion of missing data. Furthermore, we did not evaluate service-users preferences of care and/or rejection of treatment with clozapine [29], nor other potential individual barriers that could be associated with refusal of clozapine treatment, including the perception of stigma related to clozapine treatment, severe economic deprivation, which could limit access to transportation and participation on the frequent blood monitoring regime, or fear the side-effects of clozapine (e.g., weight gain) [49]. Finally, a relevant limitation is the non-inclusion of information regarding physical conditions that could affect the decision to prescribe clozapine [45]. The impact of potential bias due to the mentioned drawbacks cannot be fully estimated and future studies should address these limitations.

## Conclusions

This study reveals that Black service-users with TRS have half the odds of being treated the recommended treatment, clozapine, after accounting for several possible confounders. Thus, further research is needed to understand the drivers of ethnic inequities in access to clozapine. Reduced odds of having treatment with clozapine may place Black people at greater risk of negative outcomes, such as higher hospital readmission [15] and mortality [16].

Neutropenia, a potential driver of ethnic inequalities [29], was not associated with clozapine treatment in this study. Also, there is evidence, from this and previous studies [6–8, 15, 42, 56], to suggest that Black people with psychosis may be facing unequal care across several stages of illness care trajectory—from referral to secondary care to the third-line antipsychotic treatment, clozapine. Additionally, the negative relation between lifetime detention under the MHA and treatment with clozapine may suggest a vicious circle of poor engagement with services and not receiving the recommended care [43]. Optimal use of healthcare can be limited due to several factors [57], and to decrease healthcare inequalities, interventions taking a systemic approach should be implemented. Furthermore, it is possible that clinical decisions are being affected by unconscious bias. Actions to challenge interpersonal, institutional and structural bias in mental healthcare are recommended [53, 58, 59].

## Supplementary Information

Below is the link to the electronic supplementary material.

Supplementary file1 (DOCX 57 kb)
