# Supplementary material for: Ethnic inequalities in clozapine use among people with treatment-resistant schizophrenia: a retrospective cohort study using data from electronic clinical records
Source: Soc Psychiatry Psychiatr Epidemiol. 2022 Mar 4;57(7):1341–55. doi: 10.1007/s00127-022-02257-3 (PMC9246775; doi:10.1007/s00127-022-02257-3)
Supplement: Supplementary file 1 — Supplementary file1 (DOCX 57 kb) [file 127_2022_2257_MOESM1_ESM.docx]

**Supplementary Material**

**Table S1**

*Algorithms of treatment-resistant schizophrenia (TRS) developed and tested against a gold-standard, manually-coded, cohort of TRS*

| TRS algorithms analysed | Precision | Recall |
| --- | --- | --- |
| Three trials of treatment, or clozapine prescription, or ZTAS registry | P = 48% | R = 97% |
| Three trials of treatment with hospitalisation at any time before the initiation of the 3^rd^ antipsychotic, or clozapine prescription, or ZTAS registry | P = 52% | R = 88% |
| Three trials of treatment where the 3^rd^ antipsychotic was initiated during hospitalisation, or clozapine prescription, or ZTAS registry | P = 60% | R = 79% |
| Four trials of treatment, or clozapine prescription, or ZTAS registry | P = 62% | R = 88% |
| Four trials of treatment with hospitalisation at any time before the initiation of the 4^rd^ antipsychotic, or clozapine prescription, or ZTAS registry | P = 64% | R = 84% |
| Four trials of treatment where the 4^rd^ antipsychotic was initiated during hospitalisation, or clozapine prescription, or ZTAS registry | P = 72% | R = 75% |
| Five trials of treatment, or clozapine prescription, or ZTAS registry | P = 73% | R = 78% |
| Five trials of treatment with hospitalisation at any time before the initiation of the 5^th^ antipsychotic, or clozapine prescription, or ZTAS registry | P = 78% | R = 72% |
| Five trials of treatment where the 5^th^ antipsychotic was initiated during hospitalisation, or clozapine prescription, or ZTAS registry | P = 78% | R = 72% |
| Six trials of treatment, or clozapine prescription, or ZTAS registry | P = 84% | R = 73% |
| Six trials of treatment with hospitalisation at any time before the initiation of the 6^th^ antipsychotic, or clozapine prescription, or ZTAS registry | P = 84% | R = 73% |
| Six trials of treatment where the 6^th^ antipsychotic was initiated during hospitalisation, or clozapine prescription, or ZTAS registry | P = 86% | R = 70% |
| Seven trials of treatment, or clozapine prescription, or ZTAS registry | P = 88% | R = 70% |
| Seven trials of treatment with hospitalisation at any time before the initiation of the 7^th^ antipsychotic, or clozapine prescription, or ZTAS registry | P = 89% | R = 70% |
| Seven trials of treatment where the 7^th^ antipsychotic was initiated during hospitalisation, or clozapine prescription, or ZTAS registry | P = 88% | R = 70% |

**Table S2**

*Ethnicity and odds of clozapine prescription in a cohort of people with TRS identified before the end date of the CRIS linkage with ZTAS registry (31/03/2016); (N = 1,987)*

| **Ethnicity** | **Odds ratio** | **[95% CI]** | ***p*-value** |
| --- | --- | --- | --- |
| White British | Ref |  |  |
| Black Caribbean | 0.67 | [0.44, 1.00] | 0.053 |
| Black British / Other Black background | 0.55 | [0.36, 0.84] | 0.006 |
| Black African | 0.48 | [0.32, 074] | 0.001 |
| Other White background | 0.91 | [0.49, 1.69] | 0.756 |
| Other ethnic background | 0.56 | [0.29, 1.10] | 0.095 |
| Asian British / Other Asian background | 0.52 | [0.26, 1.05] | 0.068 |
| South Asian | 0.91 | [0.34, 2.39] | 0.846 |

The sensitivity analyses revealed that the direction and magnitude of the observed differences among Black African, Black Caribbean and Black British or other Black background were similar to the fully adjusted model. The reduced risk for treatment with clozapine among Black Caribbean, compared to White British, was no longer significant; however, given that the magnitude of association was very similar, while the sample size was reduced, we assume this was due to reduced statistical power in this analysis.
